# Venom Peptide Toxins Targeting the Outer Pore Region of Transient Receptor Potential Vanilloid 1 in Pain: Implications for Analgesic Drug Development

**DOI:** 10.3390/ijms23105772

**Published:** 2022-05-21

**Authors:** Sung-Min Hwang, Youn-Yi Jo, Cinder Faith Cohen, Yong-Ho Kim, Temugin Berta, Chul-Kyu Park

**Affiliations:** 1 Gachon Pain Center and Department of Physiology, Gachon University College of Medicine, Incheon 21999, Korea; unclehwang76@gmail.com (S.-M.H.); euro16@gachon.ac.kr (Y.-H.K.); 2 Gil Medical Center, Department of Anesthesiology and Pain Medicine, Gachon University, Incheon 21565, Korea; endless37@gilhospital.com; 3 Pain Research Center, Department of Anesthesiology, University of Cincinnati Medical Center, Cincinnati, OH 45242, USA; cohencf@mail.uc.edu

**Keywords:** transient receptor potential vanilloid 1, pain, venom, peptide, ligand

## Abstract

The transient receptor potential vanilloid 1 (TRPV1) ion channel plays an important role in the peripheral nociceptive pathway. TRPV1 is a polymodal receptor that can be activated by multiple types of ligands and painful stimuli, such as noxious heat and protons, and contributes to various acute and chronic pain conditions. Therefore, TRPV1 is emerging as a novel therapeutic target for the treatment of various pain conditions. Notably, various peptides isolated from venomous animals potently and selectively control the activation and inhibition of TRPV1 by binding to its outer pore region. This review will focus on the mechanisms by which venom-derived peptides interact with this portion of TRPV1 to control receptor functions and how these mechanisms can drive the development of new types of analgesics.

## 1. Introduction

The transient receptor potential vanilloid 1 (TRPV1) channel is a ligand-gated and non-selective cation channel that mediates physiological and pathophysiological functions in the peripheral nervous system [1,2]. TRPV1 is expressed in peripheral sensory neurons along with important signaling complexes that are associated with nociceptive mechanisms underlying various pain symptoms [3,4]. TRPV1 is activated by various noxious stimuli, such as heat, protons, plant and animal toxins, and bioactive lipids [1,5,6,7,8,9]. The different TRPV1 activation profiles elicited by different stimuli have been suggested to cause diverse pain sensations [7]. For example, endovanilloids are associated with inflammatory pain and hypersensitivity, while the exovanilloid toxin capsaicin causes acute pain [9]. For this reason, the TRPV1 receptor is a promising target in the search for novel analgesic drugs and therapeutic interventions for various pain conditions [10]. TRPV1 functions as a multimodal sensor for pain production [11], and the response elicited by TRPV1 antagonists depends on different conditions that affect the activity and inhibition of this channel [3].

Venoms from animals such as snakes, scorpions, marine cone snails, jellyfish, insects, and spiders are diverse and complex compounds that contain small molecules, peptides, and proteins [12,13]. This complex mixture can act on receptors or channels, causing irritation, paralysis, or pain [14,15]. While crude venom causes painful sensations through various mechanisms, some isolated toxins can promote analgesia by reducing nociceptive transmission [16,17,18,19]. Although the mechanism by which these toxins modulate pain remains elusive, multiple peptide toxins have been shown to act as ligands for various receptors and ion channels in the nervous system [13,20]. These peptides are stabilized by the formation of multiple disulfide bridges, which induce compact folding and are small in size, resulting in high thermal and chemical stability [21,22]. Therefore, these isolated peptides have become useful and potent pharmacological tools for investigating the structure and function of specific ion channels [13,23,24]. Recently, several venom-derived peptides have been reported to function as agonists or antagonists that bind to specific TRPV1 sites [6,25]. Therefore, these peptides can be considered important templates for the study of the structure and function of TRPV1 activity and the discovery of new analgesics.

Here, we present a brief summary of the venom-mediated modulation of TRPV1 function, focusing on peptides that specifically interact with the outer pore domain of TRPV1, with the purpose of elucidating TRPV1-mediated pain mechanisms. 

## 2. General Characteristics of TRPV1

### 2.1. The TRP Superfamily and TRPV Family

Mammalian TRP channels comprise 28 members and are divided into six subfamilies based on sequence homology: canonical (TRPC), vanilloid (TRPV), ankyrin (TRPA), melastatin (TRPM), polycystin (TRPP), and mucolipin (TRPML) [1,2,3]. Most TRP channels belong to a large family group of non-selective and polymodal ion channels with diverse physiological functions and regulatory mechanisms and are implicated in different channelopathies [26,27,28]. TRPs are tetrameric channels formed by monomers with six transmembrane domains (S1–6) and cation-selective pores [26,29,30]. 

The TRPV family has a structure similar to that of other TRP channels, but with an additional three to five ankyrin repeat domains at the N-terminal end [31]. In addition, the TRPV family consists of six vanilloid members (TRPV1–6) with different functional features [29,32]. The TRPV1–4 channels form both homo- and heterodimers with mild Ca^2+^ selectivity, while TRPV5 and 6 are highly selective for Ca^2+^ [29,33,34]. Among the TRPV homologs, the TRPV1 channel has been particularly studied with regard to its role in nociception, and, as such, is the treatment target for various pain sensations [27,29,32]. Therefore, various compounds have been developed to downregulate or inactivate the activity of TRPV1 [29,33,34,35].

### 2.2. The Structure of TRPV1 Channel

**Figure 1:**
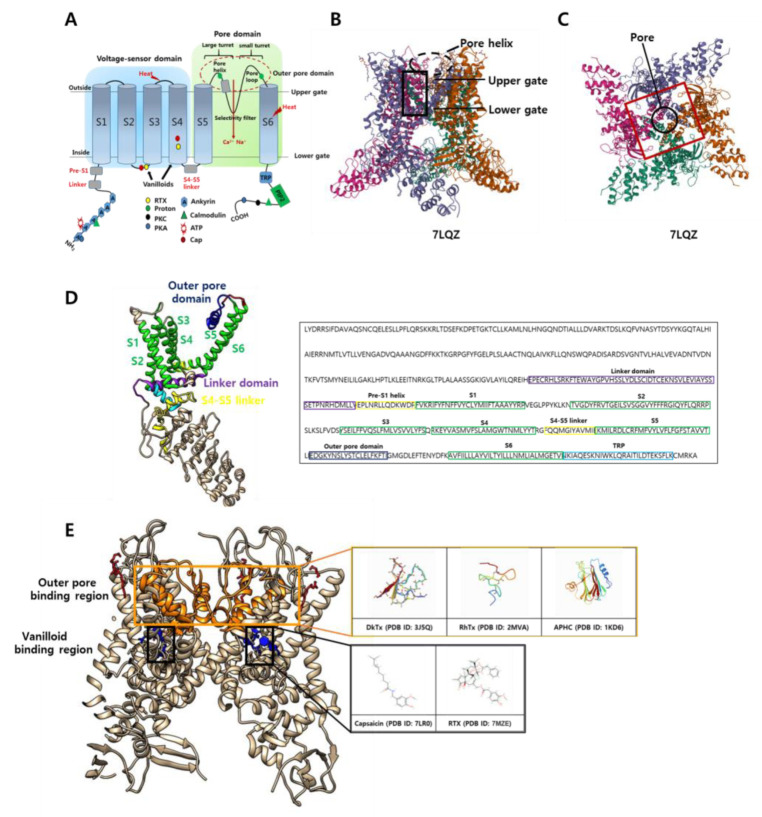
The structure and binding sites of the transient receptor potential vanilloid 1 (TRPV1) channel. (**A**) TRPV1 is a homotetrameric transmembrane protein containing a voltage-sensory domain, pore domain, S4–5 helix linker, and TRP box. The N-terminal end comprises ankyrin repeats and a calmodulin interaction site, while the C-terminal end contains a PIP2 interaction site. Amino acid residues at both ends can be phosphorylated by PKC and PKA. Vanilloid agonist sites are located in the S2–4 transmembrane domain. Both heat- and protein-initiated stimuli are mediated by specific residues located in the extracellular membrane domain (or loops). The selectivity filter is formed by the loop connecting the pore helix and S6 helix. The large and small turret subunits are connected to the S5 and S6 domains. The outer pore domain is indicated by a red dashed circle. (**B**) Side view of the TRPV1 structure: the tetrameric structure of the pore helix and the upper and lower gates, which regulate channel activation. The vanilloid pocket region is highlighted by the black box, and the pore helix is indicated by a black dashed circle (PBD ID: 7LQZ). (**C**) Top view of the TRPV1 structure: the outer pore region is highlighted by a red box, and the pore region is indicated by a black circle (PBD ID: 7LQZ). The symbols beneath each 3D structure are the access numbers in the Protein Data Bank (PDB). (**D**) Side view of a single TRPV1 subunit color coded as described in B (PDB ID: 7L2S). Sequence alignment of rat TRPV1 construct (NW_024405602); linker domain, pre-S1 helix (linker), S1–6, outer pore domain, and TRP are highlighted in purple, yellow, green, dark blue, and light blue, respectively. (**E**) Side view of the TRPV1 tetrameric structure (PDB ID: 7L2S) showing the outer pore binding venom peptides (DkTx, RhTx, and APHC) and their sites (orange box). Similarly, vanilloid binding agonist (capsaicin and RTX) and its site (black box) are shown.

TRPV1 is a homotetramer, where each homomeric subunit consists of six intracellular transmembrane core regions (S1–6) with a hydrophobic stretch (pore domain) between S5 and S6 (Figure 1) [36]. S1–4 are considered to contain a voltage sensor, an N-terminal ankyrin repeat, a linker, a pre-S1 linker, and a binding site for various agonists [1]. TRPV1 is structurally similar to the voltage-gated potassium (Kv) channel with a linker region between S4 and S5 that plays an important role in the allosteric coupling between channel domains and converts the conformational changes of S1–4 into pore gating [37]. This pore-forming loop of TRPV1 contains an outer pore region, a “turret” for channel activation, and is ion-selective [1,36]. The long N-terminus contains six ankyrin repeat domains that provide several binding sites, which are necessary for the recognition of several signaling molecules, such as calmodulin, adenosine triphosphate, phosphate groups, PI(4,5)P_2_, and calmodulin for modulating TRPV1 activation [38]. The C-terminus also interacts with various proteins and ligands [1]. In particular, TRPV1 has an amphipathic helix called the TRP box near the C-terminus, which connects the S6 helix with the C-terminus domain and is required for the allosteric channel activation [39,40]. 

### 2.3. The Function of the TRPV1 Channel

Due to its expression in the peripheral and central nervous systems, the TRPV1 channel has received attention for its involvement in mediating pain signals in physiological or pathological conditions [41]. TRPV1 is also involved in many other signals unrelated to nociception [42]. In the brain, TRPV1 can modulate the regulation of synaptic transmission, plasticity, development, and microglia-to-neuron communication [43]. Thus, TRPV1 channels are critical potential detectors of harmful stimuli as well as pain biomarkers in the brain [44]. In addition, the widespread expression of TRPV1 in peripheral tissues, including epithelial cells, the gastrointestinal tract, and immune cells, has been reported to be involved in multiple important functions in non-pain-related signal transmission [45]. Antagonists of TRPV1 inhibit pain behaviors in rodent models of cancer, osteoarthritis, and inflammation [46]. 

### 2.4. The Mechanism of Heat-Dependent Opening of TRPV1

TRPV1 is responsible for sensing body temperature, eliciting an increase in body temperature (hyperthermia) [47]. The knock out of TRPV1 in mice or TRPV1 antagonists induces prolonged hyperthermia, an undesired side effect, upon exposure to warm ambient temperature, whereas this effect is not seen in wild-type mice [48]. In addition, patients have shown long-lasting increases in body temperature after the administration of a TRPV1 antagonist [48,49]. Conversely, agonists of TRPV1, such as capsaicin, induce pain and a drop in body temperature (hypothermia) [50]. Although the TRPV1 channel is known as a high-temperature sensor that is activated above 43 °C, many of the mechanisms driving thermal sensation remain unknown [1,2]. Thermal-sensing regions of TRPV1 have been proposed to include the outer pore, cytosol, and proximal regions of the membrane [3]. Whether these regions directly detect heat or indirectly detect heat by participating in gating events downstream of thermal sensation remains debatable [4]. Additionally, it is unclear how these spatially separated regions are structured for heat-dependent gate opening and if they undergo sequential conformational rearrangements or changes in temperature activation [4,5]. Recent studies of the TRPV1 structure have revealed that the heat-induced transition of TRPV1 switches from a closed to open configuration at different temperatures [6,51]. Molecular dynamics simulations have revealed the TRPV1 structure in the closed and open states at 30 °C and 60 °C [6]. In the closed state, there is a constricted stably closed channel in the lower gate (near residue I679), whereas the upper gate (near residues G643 and M644) is dynamic and undergoes opening and (or) closing.

Open-state simulations at 60 °C have shown higher conformational changes upon thermal activation, and both the lower and upper gates are dynamic, with transient opening and (or) closing [7]. Therefore, G643 is more flexible than I679 and the upper gate can be opened more readily than the lower gate during gating transition [8]. In particular, the C-terminal domain, intracellular linker, and outer domains play a role in ligand and thermal sensation [2]. The pore helices S5 and S6 form a gate that regulates ion passage in the hydrated central cavity between the intracellular and extracellular gates [2]. Asparagine (N676) in the middle of the S6 helix adopts two conformational changes in the open and closed states [52]. In the closed state, peripheral cavities host several water molecules and the pore is partially dehydrated; N676 projects the side chain toward either the pore or four peripheral cavities located between the S6 helix and the S4–5 linker [53]. The application of activating stimulus dehydrates the peripheral cavities, causing N676 to rotate, altering the hydrophobic character of the molecular surface lining the pore, which promotes hydration and ion permeation, key components of TRPV1 activation [53].

### 2.5. The TRPV1 Pore

The TRPV1 structure includes a dual-gate channel pore with two constrictions (the upper and lower gates) located near the outer pore (residues G643 and M644) [6,25,54]. The two gates form a funnel-like shape, which spans from the extracellular face of the channel to the center of the membrane, creating a cation selectivity filter [55,56]. The lower gate is located in the middle of the S6 helix (residue I679) which lines the pore [55,57]. Channel opening occurs through a large structural rearrangement of the outer pore domain, including the pore helix and selectivity filter, accompanied by an expansion of the hydrophobic constriction of the lower gate, suggesting that this dual gating system undergoes dynamic fluctuations [2,55].

## 3. Functional Regulation of TRPV1 by Venom Peptide

### 3.1. Venom Peptides

#### 3.1.1. Relevance to Pain and Analgesia

Natural venom toxins are complex mixtures of substances such as salts, small molecules, peptides, and proteins [58]. In particular, several painful venom components are neurotoxins that act directly or indirectly on specific ion channels in the peripheral sensory nervous system, thereby affecting pain sensation [12,20,59]. A wide range of peptide toxins show functional diversity in targeting ion channels and receptors involved in pain signaling pathways [23]. Furthermore, these peptides have a high potency, selectivity, and biological stability for the development of pharmacological probes and analgesics [60].

#### 3.1.2. The Inhibitor Cystine Knot Domain of Venom Peptides

Peptides and proteins found in venomous animals contain high levels of tertiary structures stabilized by disulfide bonds [20,61]. Peptides containing the inhibitor cystine knot (ICK) motif are usually 26–50 amino acids in length and exhibit various activities, including ion channel blocking [62,63]. The ubiquitous ICK motif, which is the most well-known disulfide-rich framework, is defined as an antiparallel β-sheet stabilized by a cystine knot [64,65]. The main advantages of the use of ICK peptides for drug development are their resistance to proteases, high temperature, and harsh chemicals, which are attributed to their knotted structure [66]. Recent compounds reportedly developed from venoms have included peptide toxins that contain a specific structural motif with an ICK domain [67]. 

### 3.2. TRPV1 Activation by Venom Peptides

#### 3.2.1. DkTx

The DkTx toxin, derived from the venom of the Malaysian earthtiger tarantula, binds to the extracellular pore domain of TRPV1, locking the receptor open and causing pain [68] (Table 1). DkTx consists of two ICK domains, known as K1 and K2 [69]. Each ICK domain contains six cysteine residues that form sulfide bridges (knot-like structures) [6]. DkTx can form a stable, non-covalent complex with the outer pore region of TRPV1 through the interaction between four of its residues and the S5 and S6 pore helix loops (I599 and F649, respectively) and S6 (A657 and F659, respectively) [6,70]. Furthermore, ICK peptide toxins can specifically interact with voltage-gated ion channels [71]. Thus, DkTx activates TRPV1, which belongs to the voltage-gated ion-channel superfamily [72].

#### 3.2.2. Vanillotoxins (VaTx1–3)

VaTxs are ICK motif-containing peptides isolated from the venom of the tarantula *Psalmopoeus cambridgei* [6]. VaTxs act as TRPV1 agonists, activating pain sensation by binding to the extracellular pore domain of the channel, opening the pore, and triggering the influx of cations [6,54]. VaTx1 and VaTx2 also act as antagonists of the Kv2-type voltage-gated K^+^ channel (Kv2), causing paralysis and hyperexcitability [6]. Despite the similar structures of TRPV1 and Kv2, VaTx1–2 binds to the voltage-sensing domain of Kv2 rather than the pore domain [14]. 

#### 3.2.3. RhTx

RhTx is a peptide toxin (27 amino acids) found in *Scolopendra subspinipes mutilans* (also known as the Chinese red-headed centipede) venom [79]. RhTx has two pairs of disulfide bonds: the N-terminus of the peptide contains no charged amino acids, whereas the C-terminus of the peptide is rich in charged amino acids [79,80]. Four charged residues (D20, K21, Q22, and E27) and one polar residue (R15) participate in RhTx-TRPV1 binding [89]. RhTx has a high-temperature dependence because an increase in temperature potentiates its activity; thus, RhTx is a potent TRPV1 activator at physiological body temperature, causing intense burning pain [6,80]. Specifically, RhTx binds to the outer pore region of TRPV1 as a selective TRPV1 activator and does not affect other TRPV channels (TRPV2–4) [6]. 

#### 3.2.4. BmP01

BmP01 is a short peptide (29 amino acids) isolated from the venom of the scorpion *Mesobuthus martensii* with a typical ICK motif structure [5]. This toxin has dual functions, as it both activates TRPV1 and inhibits the Kv channel, thus resulting in a hyperexcitable nociceptive condition [82]. BmP01 binds to the outer pore domain of TRPV1, which contains three polar residues located in the pore helix-S6 loop (E649, T651, and E652 of hTRPV1) that are known to affect TRPV1 gating [5,81]. Bmp01 induces pain signals in wild-type but not in TRPV1 KO mice, suggesting that this peptide toxin could produce pain through TRPV1 activation [82].

### 3.3. TRPV1 Inhibition by Venom Peptides

#### 3.3.1. Analgesic Polypeptide *Heteractis crispa* Toxin

Analgesic polypeptide *Heteractis crispa* (APHC)1–3 are 56-amino acid peptides derived from the sea anemone *Heteractis crispa* that bind to the outer loop of TRPV1 and induce analgesic activity during pain-related behavioral tests in rodents without causing hyperthermia [83,84,85]. Notably, APHCs enhance the TRPV1 response at low concentrations of capsaicin but block the response at high concentrations [83]. In addition, APHCs bind to the outer loop domain involved in proton activation [83]. This can directly affect the opening of the upper gate of TRPV1 because the binding site affects proton-binding conditions and can be allosterically coupled with APHC- or capsaicin-binding sites [83].

#### 3.3.2. *Heteractis crispa* RG 21

*Heteractis crispa RG 21* (HCRG21) is a new Kunitz-type peptide that shares a high structural homology with the Kunitz peptide (APHC)1–3 [6]. This peptide inhibits the capsaicin-induced ion current through TRPV1 [87] and results in a dramatic decrease in TRPV1-mediated tumor necrosis factor-α production in a model of carrageenan-induced pain [88]. Therefore, this new peptide has a pharmacological target as a TRPV1 antagonist. Altogether, the outer loop of TRPV1 is a crucial site to study, as various ligands that can bind to this site produce differential effects that have implications for the development of alternative pain therapeutics.

### 3.4. Complex between TRPV1 and Peptides (Toxins)

Peptide toxins can modulate TRPV1 activation by binding to specific domains, including the outer pore domain [6]. Various peptide toxins directly inhibit or activate this channel by binding to sites in the outer pore domain involved in the channel’s gating mechanism [90,91]. In particular, some toxins, including DxTx, BmP01, and RhTx, have been shown to activate or inhibit TRPV1 by binding to the outer pore domain of the channels (Figure 1) [6,90,91]. This implies that the outer pore region of TRPV1 is a common domain for binding and channel gating by different peptide animal toxins and highlights the importance of the outer pore domain in TRPV1 activation [36]. The outer pore region plays a role in TRPV1 activation because this raises the potential to allosterically modulate TRPV1 activation by other stimuli, including heat and proteins [36,92,93].

#### 3.4.1. Open and Closed State: Dual Gating Mechanism

The TRPV1 structure showcases three distinct structural states: the closed state (apo condition) is the ligand-free condition; the partially open state is the capsaicin-binding condition; and the fully open state is the binding state caused by resiniferatoxin (RTX) and double-knot toxin (DkTx), both of which act as irreversible TRPV1 channel openers [36,94]. The closed state constricts the selectivity filter and lower gate [55,95]. The two open states of TRPV1 are determined by the binding state of capsaicin or RTX/DkTx [2,5]. When capsaicin binds, there is no change in the selectivity filter, whereas the lower gate expands significantly [36,96]. In contrast, when RTX/DkTx binds, the channel is fully open to the ion conduction pathway, where all constrictions are eliminated [6,25,36,83].

#### 3.4.2. The Outer Pore Region of TRPV1

Two major TRPV1-binding sites are responsible for the open gating mechanism in response to multiple agonists and antagonists [1]. The first is a capsaicin binding site located in S3–4, and the second is an outer pore region that contains binding sites for various ligands, and, as such, is responsive to heat, protons, and venom peptide toxins [1,7,38,40,54,57,97,98]. Residues in this region are essential for the heat-mediated activation of TRPV1 [92]. Extracellular protons potentiate and activate TRPV1 through glutamate residues found in this region (E600 and E648) [99]. Furthermore, capsaicin binding induces the outward movement of the S4–5 linker, but few conformational changes occur in the outer pore region [100]. In contrast, large conformational changes occur during heat activation [94,101]. Some venom peptide toxins can allosterically modulate TRPV1 activation through the outer pore region domain [6]. RhTx activates TRPV1 through an allosteric mechanism and promotes TRPV1 opening by preferentially binding to the activated state [102]. RhTx also promotes heat activation by lowering the activation threshold temperature [102].

#### 3.4.3. The Allosteric Coupling of TRPV1: Upper and Lower Gates

As the TRPV1 channel is key in the pain pathway, blocking TRPV1 activation could constitute a novel strategy to alleviate various pain sensations [95]. Allosteric conformational changes of TRPV1 are an interesting mechanism, as various vanilloid and venom peptide toxins interact with specific binding domains to affect the upper and lower gates of the open state of TRPV1 [103]. In the absence of an activating stimulus, both gates of TRPV1 remain closed, whereas the binding of an activating ligand causes both gates to partially or sequentially open [83]. For example, vanilloid agonists (vanillyl group) and capsaicin interact with the S4–5 linker of TRPV1 to induce conformational rearrangements (outward movement of the S4–5 linker) that are concomitant with the opening of the lower gate and result in small conformational changes in the outer pore region [73]. RhTx directly interacts with the outer pore helix and turret of TRPV1, which produces excruciating pain [79]. DkTx also binds to the outer binding domain of TRPV1, stabilizing the open state and evoking irreversible channel activation [54]. Notably, capsaicin and DkTx initiate different gating mechanisms through different binding domains within the TRPV1 pore turret domain [54]. Therefore, a difference in the movement of the outer pore region may be exploited to develop a modality-specific inhibitor of TRPV1 without eliciting adverse effects on body temperature and thermal sensation. 

#### 3.4.4. The Two Distinct Binding Sites of TRPV1

TRPV1 is a non-selective cation channel that is activated by various specific endogenous and exogenous ligands [56]. TRPV1 activation leads to painful burning sensations [1,55,104]. Exogenous agonists include dietary compounds (capsaicin and eugenol), non-dietary plant compounds (RTX), animal venoms, and other factors (noxious heat and extracellular pH) [38,105]. Endogenous agonists include bioactive lipids, inflammatory soups (bradykinin and histamine), and mediators of inflammation (LOX, a product of the lipoxygenases) [1,4,38,95]. These activators bind to the binding sites of the extracellular and intracellular domains of TRPV1, producing distinct pain sensations [2,36,95,97].

Notably, these binding sites are located in separate regions within the TRPV1 channel subunits and lead to various pain sensations [2,9]. For example, DkTx interacts with the outer pore domain and produces persistent pain through prolonged TRPV1 activation, whereas capsaicin, a key exogenous ligand for TRPV1, evokes a sensation of heat and acute pain [70,106]. Capsaicin can permeate the plasma membrane and bind in a “tail-up, head-down” orientation to the capsaicin-binding (vanilloid binding) pocket region formed by the S3, S4, and S4–5 linkers of TRPV1 [2,5,75]. Similarly, RTX, an ultrapotent agonist of TRPV1, binds to a location close to Y511 and S512 in S3 and M547 in S4 [36]. In addition, extracellular protons are important physiological stimulators of TRPV1 [92]. Hydrogen-binding sites are located in the outer pore domain of TRPV1 and potentiate the heat sensitivity of TRPV1 [92]. In addition to plant-derived TRPV1 agonists, various animal-derived toxins have been reported to act on the pore region of TRPV1. These act directly or indirectly by stimulating pathways that can sensitize TRPV1 [56,81]. The mechanisms underlying the various pain sensations mediated by TRPV1 are not fully understood. Therefore, investigating the mechanism of TRPV1 activation and the inhibition of both the intracellular vanilloid-binding site and outer pore domain site is important for the discovery of pain inhibitors.

## 4. Functional Regulation of TRPV1 as a Venom Peptide Target

### 4.1. Side Effects of TRPV1 Inhibition as a Drug Target

The diverse physiological roles of TRPV1 pose a serious challenge to drug development, notably for obtaining sufficient specificity for clinically useful interventions without undesirable side effects, such as burns and hyperthermia [107]. As TRPV1 is a multimodal ion channel that responds to several stimuli, including heat, low pH, and capsaicin [11], the development of a modality-dependent inhibitor is necessary. For example, antagonists that do not inhibit the thermal activation of TRPV1 do not induce hyperthermia [25]. These inhibitors may still have beneficial effects by blocking other specific receptor modalities activated by low pH or other agonists 108]. Thus, antagonists that selectively block the sensitized state of the channel without blocking basal activity may be free of these side effects [108]. TRPV1 is unique among drug targets because the initial agonist-induced burning sensation is followed by sustained desensitization [109]. Therefore, although capsaicin evokes an initial pain reaction, desensitization to the compound has therapeutic potential as a functional antagonist of TRPV1 [107]. The efficacy of TRPV1 agonists or antagonists in preclinical pain models varies [25,55,107,110,111]. However, different effects in inflammatory and neuropathic pain models have been observed, implying that the TRPV1 targets for pain relief are more diverse [104,111,112,113].

### 4.2. Minimizing Side Effects of TRPV1 Inhibition as a Drug Target

The magnitude of the analgesic efficacy of TRPV1 activation depends on the various stimuli used [83,114]. TRPV1 is highly unusual in that it can modulate similar or different mechanisms, leading to its antagonists having both hyper- and hypothermic effects [115]. Unlike most TRPV1 antagonists, APHC1 and APHC3 exhibit significant analgesic activity in vivo at a low dose, which causes a moderate decrease in core body temperature [85]. More specifically, APHC1 and APHC3 are highly homologous and differ in 4 of 56 amino acids [95]. However, only APHC3 can inhibit the low-pH-mediated activation of TRPV1 [85,95]. The outer loop domain site at which TRPV1 is bound by these polypeptides (APHC1–3) can affect activation by protons and can be allosterically bound to the capsaicin site [83,86]. In addition, other polypeptides also interact with the outer loop domain of TRPV1 and mediate the pain response [6,80,82]. Therefore, these polypeptide toxins could be used as a new class of TRPV1 modulators with significant analgesic effects and without side effects such as hyperthermia, as well as in drug design templates for pain inhibition.

## 5. Conclusions

TRPV1 is a polymodal receptor activated by endo- and exoligands, heat, lipids, and voltage, with highly distinct functions not only in the nervous system but also in peripheral tissues. Thus, TRPV1 has become an attractive target for the development of novel pain inhibitors, and several inhibitors of TRPV1 have been developed as new analgesics. However, most of these failed testing during clinical trials in animals and humans owing to their serious side effects, such as hyperthermia and altered thermal sensation. Thus, it is necessary to find TRPV1 antagonists that precisely regulate TRPV1 without adverse effects. The venom peptide is a bioactive component that has been reported to probe pain regulatory mechanisms through various ion channels, including TRPV1. However, despite extensive knowledge concerning the limited number of disulfide structures present in animal venom, numerous families of disulfide-rich venom peptides have not been explored. Therefore, this review described the limitations and potential benefits of studying allosteric coupling between the dual gating mechanism (upper and lower gates) of TRPV1 using peptide toxins targeting the outer pore region of TRPV1 that integrate diverse pain signals from TRPV1 activation. In conclusion, the studies evaluated show that it is possible to develop key pain-related modality-dependent inhibitors of TRPV1 using specific venom peptides.

## Figures and Tables

**Table 1:** Various ligands and venom peptides (activators/inhibitors) of the transient receptor potential vanilloid 1 (TRPV1) channel.

Ligands	Binding Region	Binding Type	Pain Condition	Reference
Capsaicin	Vanilloid-binding pocket	Hydrogen, Van der Waals bond	Pain sensation	[73,74,75]
RTX	Vanilloid-binding pocket	Hydrogen bond	Pain sensation	[73,76]
Proton	Outer pore domain	Hydrogen bond	Pain sensation	[36]
Double-knot toxin (DkTx)	Outer pore domain	Hydrophobic interaction	Pain sensation	[6,54,68,70,71,77,78]
Vanillotoxins (VaTx1–3)	Outer pore domain	Electrostatic interaction	Pain sensation	[5,6,14]
Scolopendra subspinipes mutilans toxin RhTx	Outer pore domain	Electrostatic and hydrophobic interactions	Pain sensation	[2,79,80]
Pain-inducing peptide (BmP01)	Outer pore domain	Electrostatic interaction	Pain sensation	[5,81,82]
Analgesic polypeptide *Heteractis crispa* (APHC) toxin	Outer pore domain	No direct evidence	Analgesic action	[83,84,85,86]
*Heteractis crispa* RG 21 (HCRG21)	Outer pore domain	No direct evidence	Analgesic action	[87,88]
